# Knowledge, Attitude, and Practice (KAP) toward COVID-19 Pandemic among the Public in Taiwan: A Cross-Sectional Study

**DOI:** 10.3390/ijerph19052784

**Published:** 2022-02-27

**Authors:** Yi-Fang Luo, Liang-Ching Chen, Shu-Ching Yang, Shinhye Hong

**Affiliations:** 1 Center for Teaching and Learning Development, National Kaohsiung University of Science and Technology, Kaohsiung 805301, Taiwan; a0989909301@gmail.com; 2 Intelligent Electronic Commerce Research Center, Institute of Education, National Sun Yat-sen University, Kaohsiung 80424, Taiwan; jokiceman@gmail.com; 3 Department of Foreign Languages, R.O.C. Military Academy, Kaohsiung 83059, Taiwan

**Keywords:** knowledge, attitude, and practice (KAP), COVID-19 knowledge, affective attitude, preventive behavior

## Abstract

*Purpose:* Knowledge, attitude, and practice (KAP) models are often used by researchers in the field of public health to explore people’s healthy behaviors. Therefore, this study mainly explored the relationships among participants’ sociodemographic status, COVID-19 knowledge, affective attitudes, and preventive behaviors. *Method:* This study adopted an online survey, involving a total of 136 males and 204 females, and used a cross-sectional study to investigate the relationships between variables including gender, age, COVID-19 knowledge, positive affective attitudes (emotional wellbeing, psychological wellbeing, and social wellbeing), negative affective attitudes (negative self-perception and negative perceptions of life), and preventive behaviors (hygiene habits, reducing public activities, and helping others to prevent the epidemic). *Results:* The majority of participants in the study were knowledgeable about COVID-19. The mean COVID-19 knowledge score was 12.86 (*SD* = 1.34, range: 7–15 with a full score of 15), indicating a high level of knowledge. However, the key to decide whether participants adopt COVID-19 preventive behaviors was mainly their affective attitudes, especially positive affective attitudes (*β* = 0.18–0.25, *p* *<* 0.01), rather than COVID-19 disease knowledge (*β* = −0.01–0.08, *p* > 0.05). In addition, the sociodemographic status of the participants revealed obvious differences in the preventive behaviors; females had better preventive behaviors than males such as cooperating with the epidemic prevention hygiene habits (*t* = −5.08, *p* *<* 0.01), reducing public activities (*t* = −3.00, *p* *<* 0.01), and helping others to prevent the epidemic (*t* = −1.97, *p* *<* 0.05), while the older participants were more inclined to adopt preventive behaviors including epidemic prevention hygiene habits (*β* = 0.18, *p* = 0.001, *R*^2^ = 0.03), reducing public activities (*β* = 0.35, *p* *<* 0.001, *R*^2^ = 0.13), and helping others to prevent the epidemic (*β* = 0.27, *p* *<* 0.001, *R*^2^ = 0.07). *Conclusions:* Having adequate COVID-19 knowledge was not linked to higher involvement in precautionary behaviors. Attitudes toward COVID-19 may play a more critical function in prompting individuals to undertake preventive behaviors, and different positive affective attitudes had different predictive relationships with preventive behaviors.

## 1. Introduction

Since December 2019, COVID-19 has caused a global pandemic and seriously impacted various fields such as medicine, public health, economy, and the environment. According to the World Health Organization (WHO), with the rise of the Delta and Omicron variants [1], the number of global confirmed cases approached 300 million with 5.5 million deaths at the beginning of 2022 [2]. Large-scale vaccination has become one of the most important public health policies for countries to prevent the spread of COVID-19; however, breakthrough infections caused by the variants confirm that high vaccine coverage may not guarantee effective control of the spread of COVID-19 [3,4]. In other words, vaccination is not the only reliable way to control the pandemic, as the implementation of epidemic prevention and control measures cannot be ignored. Continuously disseminating educational propaganda to the public for them to take correct preventive behaviors such as maintaining social distance and proper hygiene habits is essential for epidemic prevention and control [5,6,7]. Thus, identifying personal preventive health behavior (PHB)-related factors is an extremely important issue amid the COVID-19 pandemic [5].

The WHO [8] pointed out that using knowledge, attitude, and practice (KAP) models to conduct studies and surveys can help in collecting information on the knowledge, opinion, attitude, and behavioral practice of relevant specific groups on a specific issue, so as to understand the relationships among knowledge, attitude, and behavior, and they can further be used as evaluation references of related programs or interventions. Hence, many studies also explored public perceptions and behaviors toward COVID-19 using KAP-based models [9,10,11]. Currently, KAP models are often used in public health research to explore people’s health behaviors and explain their changes, which can be divided into three aspects: acquiring correct knowledge, generating attitude, and adopting behavior. In addition, advocating knowledge is the basis of behavior, while attitude is the momentum of behavior, urging individuals to implement behaviors to achieve goals [12,13]. 

### 1.1. Knowledge and Behavior

Lunsford et al. [14] mentioned that providing knowledge about diseases to educate the public is considered as a strategy to help people adopt preventive health behaviors. Therefore, the knowledge of disease and health is regarded as the key to whether an individual adopts healthy behaviors, and it is also believed that the more health knowledge an individual has, the better healthy behaviors they will adopt [15,16]. From the perspective of health literacy, the acquisition and the understanding of health or disease knowledge are essential for effective self-management of health or disease [17]. In addition, people’s health literacy plays an important role in understanding specific health knowledge. That is, if they are able to understand the facts of COVID-19, they will convert the acquired knowledge into practical health-promoting behaviors to improve or maintain their health conditions [18]. However, knowledge does not necessarily elicit behavioral responses; Allegrante, Auld, and Natarajan [19] pointed out a huge gap between respondents’ knowledge and behaviors about COVID-19, whereby respondents did not take preventive health behaviors against COVID-19 not because of an insufficient knowledge of COVID-19 but due to other factors.

### 1.2. Attitude and Behavior

Liu, Teng, and Han [20] mentioned that knowledge may not be enough or the only factor inducing behavior. The influence of knowledge on related behavior was indirect, and there were other moderating variables between them. Attitude was one of them. In addition to knowledge, attitude also guides individuals’ behaviors and becomes the motivation for behavior, which prompts individuals’ intention to take a certain action [21]. Attitude is an individual’s general and persistent assessment of an object, and this assessment is multidimensional, which is usually divided into two aspects: cognitive and affective [22]. Cognitive attitude (i.e., instrumental attitude) refers to the values and beliefs related to objects or beliefs about the costs and benefits on actions, while affective attitude refers to the emotional experience related to objects, which is an emotion-based perception [23,24,25]. Emotions include moods and feelings, embracing both positive and negative emotions. When an individual feels good, the emotional state is positive; otherwise, it is negative [26,27,28].

Many studies on attitude and behavior focused on the cognitive aspect of attitude, but less on the aspects of emotion [29]. For example, Alahdal, Basingab, and Alotaibi [30] indicated that there was significant positive correlation between participants’ cognitive attitudes and COVID-19 preventive behaviors (i.e., beliefs that taking certain preventive actions can help to curb the spread of the disease). In KAP studies on COVID-19, the attitude assessments mainly focused on beliefs of anti-pandemic behaviors, for example, beliefs that “the pandemic will eventually be controlled” and “handwashing is essential for protecting yourself from infection” [9,11]. Moreover, prior studies integrally used cognitive and affective attitudes without distinguishing them for discussion. Pal et al. [10] researched the aspect of COVID-19 attitude; they not only evaluated individuals’ cognitive beliefs about anti-pandemic behaviors, but also included emotional evaluations such as COVID-19 anxiety. However, other studies pointed out that cognitive and affective attitudes are two independent aspects; thus, it is necessary to distinguish them [24,27].

Due to the limitations of current research in explaining affective attitudes with respect to behaviors, it is necessary to add variables of affective attitude as an extension of behavior-related theories [29]. Therefore, the main purpose of this study was to understand the current status of the Taiwanese public’s COVID-19 knowledge, attitude, and behavior. Furthermore, sociodemographic status may have an effect on personal preventive measures amid the COVID-19 pandemic [7]; thus, this study also explores the relationship between sociodemographic status (e.g., gender and age) and KAP of COVID-19. Lastly, the relationships among COVID-19 knowledge, attitudes, and behaviors of the Taiwanese public are also analyzed. The attitude element focuses on the emotional aspect to compensate for the research gaps in prior studies.

## 2. Methodology

### 2.1. Participants

A total of 354 respondents were recruited from different regions of Taiwan through the internet to complete online anonymous questionnaires. After removing the incomplete data, the valid questionnaires (*n* = 340) were processed for further statistical analysis (see Table 1).

### 2.2. Procedures

Regarding the implementation procedures, as the Taiwan government issued public health policies during the pandemic, this study used an online anonymous questionnaire via SurveyCake to avoid interpersonal contact. The survey period was from 17 May to 11 June 2020, before the severe COVID-19 outbreak (i.e., level 3 alert) in Taiwan (level 3 alert was from late May to July 2021). During the survey time, although the vaccine coverage was low, the government successfully controlled the spread of the epidemic through various anti-epidemic measures.

An anonymous online questionnaire was designed in traditional Chinese language to invite potential respondents. We issued the URL of the online questionnaire on social media platforms (e.g., Plurk and Facebook) and invited people to browse information freely. Instructions of the questionnaire on the front page clearly informed the participants of the research purposes and their rights regarding joining or dropping out of this study at any time during participation. All participants were informed and assured that their participation was voluntary, anonymous, and strictly confidential; moreover, they could stop participating in the study at any time without fear of penalty. The participants who did not complete the survey were excluded from the final sample.

### 2.3. Instruments

#### 2.3.1. COVID-19 Knowledge Scale

To find out whether participants had a certain level of COVID-19 knowledge, the researchers selected proper information from the section of disease information on the Taiwan Centers for Disease Control (TCDC) website [31], and then a COVID-19 knowledge scale was compiled with answers in the form of true/false and one point for each correct answer. After the content and the validity of the scale were checked by scholars and experts, we took the top 27% of the total score as the high group and the bottom 27% as the low group, before using item analysis to conduct a discrimination test. The results showed that the *t*-values of the 15 items ranged from 2.71 to 7.40, and each item met the standard (i.e., *p* < 0.05), indicating that the quality of the items was reasonable.

#### 2.3.2. COVID-19 Positive Affective Attitude Scale

This study took the COVID-19 pandemic period as the attitude object, using the items of the Mental Health Continuum Short Form (MHC-SF) [32,33] to develop the COVID-19 positive emotion attitude scale. The scale was divided into three dimensions: “emotional wellbeing” used to investigate the participants’ positive emotions and satisfaction with life amid the COVID-19 pandemic (embracing three items, Cronbach’s alpha = 0.91), “psychological wellbeing” including the participants’ perceptions of self-acceptance, personal growth, purpose in life, environmental mastery, autonomy, and positive relations with others amid the pandemic (embracing six items, Cronbach’s alpha = 0.90), and “social wellbeing” used to investigate the participants’ attitudes toward social acceptance, social actualization (i.e., social growth), social contribution, and social integration amid the pandemic (embracing five items, Cronbach’s alpha = 0.82). The scale adopted a Likert five-point scoring method. Higher scores on this scale represented greater happiness in this dimension. The overall reliability of this scale according to Cronbach’s alpha was 0.90, revealing high internal consistency.

#### 2.3.3. COVID-19 Negative Affective Attitude Scale

The researchers considered COVID-19 scenarios and referred to the Center of Epidemiology Study Depression Scale [34] to compile the COVID-19 negative affective attitude scale. The scale adopted a Likert five-point scoring method. Higher scores on this scale represented negative feelings in this dimension. The Kaiser–Meyer–Olkin (KMO) test scored higher than 0.90, and Bartlett’s test also reached significance, indicating that the scale incorporated common factors and was suitable for factor analysis. After extraction by principal component and exploratory factor analysis (EFA) with rotation by varimax, Table 2 presents the correlation commonality, factor loading, explained variations, and reliability (overall reliability = 0.88), revealing high internal consistency.

#### 2.3.4. COVID-19 Preventive Behavior Scale

This paper referred to relevant studies [35,36,37] and invited experts to inspect the questionnaires to compile the COVID-19 preventive behavior scale. The KMO test scored higher than 0.90, and Bartlett’s test also reached significance, indicating that the scale was suitable for factor analysis. Using principal component analysis and EFA with rotation by varimax, three factors were extracted: (1) willingness to cooperate with epidemic prevention regulations declared by units of the government and schools, and to integrate epidemic prevention and hygiene measures into practice; (2) cooperation with “reducing public activity” to reduce the chances of interpersonal interactions; (3) demonstration of altruistic spirit and willingness to participate in society to “help others to prevent the epidemic”. This scale adopted a Likert five-point scoring method. Higher scores on this scale represented being more proactive in response to COVID-19 in this dimension. Correlation commonality, factor loading, explained variations, and reliability are shown in Table 3.

### 2.4. Data Processing and Analysis

All analysis was performed using SPSS 22.0 software (IBM Corp, Armonk, NY, USA). The data collected online was used to describe the current situation of participants’ COVID-19 knowledge, positive and negative affective attitudes, and preventive behaviors in the form of arithmetic means and standard deviations (*SD*). First, we used the dependent sample *t*-test or multivariate analysis to test the divergences in different dimensions of each variable to address the first research purpose. Then, we used the independent sample *t*-test, regression analysis, and variance analysis to test the differences in knowledge, positive and negative affective attitudes, and preventive behavior variables as a function of different sociodemographic status variables (gender, age) to address the second research purpose. Lastly, we used hierarchical regression analysis to examine the predictive effects of COVID-19 knowledge and positive and negative affective attitudes on preventive behavior under the control of sociodemographic status to address the third research purpose.

## 3. Results

### 3.1. Current Status of COVID-19 Knowledge, Affective Attitude, and Preventive Behavior 

#### 3.1.1. COVID-19 Knowledge

The maximum score for COVID-19 knowledge questions was 15 points. The average score of participants was 12.86 points (*SD* = 1.34), with a range of 7–15 points. Among them, 36 people (10.59%) got full marks, and more than half of the participants (212 people, 62.35%) got 13 points or higher. This showed that the questions were generally easy for the participants, and their COVID-19 knowledge was good. Table 4 indicates that all participants understood that COVID-19 can be transmitted from person to person (question 2). The item with the lowest correctness rate was “the coronavirus is easy to be isolated by tissue culture” (question 6), which is understandable because a slight background knowledge of biology or medicine was necessary.

#### 3.1.2. COVID-19 Affective Attitude

Multivariate analysis of positive affective attitudes in different dimensions found that the participants exhibited significant differences (*F*_(2,338)_ = 228.46, *p <* 0.001). Scheffé’s post hoc comparison indicated that the participants’ emotional wellbeing (*M* = 2.35, *SD =* 0.94) was not only lower than the median, but also significantly lower than social wellbeing (*M* = 3.06, *SD =* 0.80) and psychological wellbeing (*M* = 3.62, *SD =* 0.71), while social wellbeing was significantly lower than psychological wellbeing. This showed that the participants’ personal positive emotion and sense of satisfaction with life were the lowest amid the COVID-19 pandemic; nevertheless, when facing a new lifestyle in the pandemic era, participants were able to achieve self-acceptance, pursue personal growth, control their purpose in life, master their environment, and maintain autonomy and positive relations with others.

On the other hand, participants’ negative attitudes toward themselves (*M* = 2.18, *SD* = 0.75) and life (*M* = 2.87, *SD* = 0.77) were lower than the mean value, indicating a low prevalence of these attitudes during the survey period. 

The paired-sample *t*-test revealed significant differences in the negative affective attitudes of different aspects (*t*_(339)_ = *−*16.66, *p* *<* 0.001), with negative attitudes toward life being significantly higher than those toward self, indicating a greater effect of the external living environment brought about by the pandemic than the individuals’ inner psychology.

#### 3.1.3. COVID-19 Preventive Behaviors

Preventive behaviors taken by the participants during the period of epidemic prevention revealed higher scores of epidemic prevention hygiene habits (*M* = 4.30, *SD* = 0.61), helping others to prevent the epidemic (*M* = 3.93, *SD* = 0.78), and reducing public activities (*M* = 3.65, *SD* = 0.87) than the median value. Multivariate analysis showed significant differences in terms of epidemic prevention behaviors of different aspects (*F*_(2,338)_ = 134.44, *p* *<* 0.001). Scheffé’s post hoc comparison indicated that participants’ hygiene habits for epidemic prevention were significantly more prevalent than helping others to prevent the epidemic and reducing public activities, with the former being more prevalent than the latter. It can be seen that the participants were generally willing to change their COVID-19 preventive behaviors, especially in terms of hygiene habits for epidemic prevention.

### 3.2. Differences between Sociodemographic Status and COVID-19 Knowledge, Affective Attitude, and Preventive Behavior

#### 3.2.1. Gender

Table 5 shows that there was no significant difference in scores in terms of gender for COVID-19 knowledge (*t* = 0.96, *p* = 0.34); the average knowledge scores were 12.94 points (*SD* = 1.30) for males and 12.80 points (*SD* = 1.37) for females. In terms of positive affective attitudes, there were no significant differences in terms of gender for emotional wellbeing (*t* = 0.98, *p* = 0.33), psychological wellbeing (*t* = *−*0.65, *p* = 0.51), and social wellbeing (*t* = 0.06, *p* = 0.95) (emotional wellbeing: *M*_(male)_ = 2.41, *SD*_(male)_ = 0.91, *M*_(female)_ = 2.31, *SD*_(female)_ = 0.96; psychological wellbeing: *M*_(male)_ = 3.59, *SD*_(male)_ = 0.77, *M*_(female)_ = 3.64, *SD*_(female)_ = 0.67; social wellbeing: *M*_(male)_ = 3.06, *SD*_(male)_ = 0.80, *M*_(female)_ = 3.06, *SD*_(female)_ = 0.80). In addition, there were also no significant differences in terms of gender for negative affective attitudes including negative self-perception (*t* = 1.16, *p* = 0.25, *M*_(male)_ = 2.23, *SD*_(male)_ = 0.80, *M*_(female)_ = 2.14, *SD*_(female)_ = 0.72) and negative perception of life (*t* = *−*1.05, *p* = 0.13, *M*_(male)_ = 2.79, *SD*_(male)_ = 0.76, *M*_(female)_ = 2.92, *SD*_(female)_ = 0.77).

There were significant differences in terms of gender for COVID-19 preventive behaviors such as cooperating with epidemic prevention hygiene habits (*t* = *−*5.08, *p* *<* 0.01), reducing public activities (*t* = *−*3.00, *p* *<* 0.01), and helping others to prevent the epidemic (*t* = *−*1.97, *p* *<* 0.05), with females being significantly more compliant (epidemic prevention hygiene habits: *M*_(male)_ = 4.10, *SD*_(male)_ = 0.65, *M*_(female)_ = 4.43, *SD*_(female)_ = 0.54; reducing public activities: *M*_(male)_ = 3.48, *SD*_(male)_ = 0.89, *M*_(female)_ = 3.76, *SD*_(female)_ = 0.84; helping others to prevent the epidemic: *M*_(male)_ = 3.82, *SD*_(male)_ = 0.76, *M*_(female)_ = 3.99, *SD*_(female)_ = 0.79).

#### 3.2.2. Age

The regression analysis results revealed no significant correlation between age and COVID-19 knowledge (*β* = 0.11, *p* = 0.054, *R*^2^ = 0.01). In terms of positive affective attitudes, age had a negative predictive relationship with emotional wellbeing (*β* = *−*0.18, *p* = 0.001, *R*^2^ = 0.03) and a positive predictive relationship with psychological wellbeing (*β* = 0.16, *p* = 0.002, *R*^2^ = 0.03), indicating that older age was correlated with lower life satisfaction amid the pandemic. Nevertheless, older participants were more capable of self-acceptance, pursuit of personal growth, mastery of life goals and the environment, and maintenance of autonomy and positive interpersonal relationships. 

Notably, the effect size *R*^2^ of age to emotional wellbeing and to psychological wellbeing was very small, indicating that, although it was statistically significant, the effects of age on these aspects of wellbeing were actually very small. On the other hand, age had no significant predictive relationship with social wellbeing (*β* = 0.003, *p* = 0.96, *R*^2^ *<* 0.001). In addition, age had negative relationships with negative self-perceptions of negative affective attitudes (*β* = −0.13, *p* = 0.02, *R*^2^ = 0.02) and negative perceptions of life (*β* = −0.12, *p* = 0.03, *R*^2^ = 0.01), showing that younger participants were more inclined to have negative affective attitudes. However, the effect size *R*^2^ was also very small, indicating that age had a very small impact on these negative affective attitudes.

This study also discovered that the older the participants, they were more likely to adopt COVID-19 preventive behaviors including the epidemic prevention hygiene habits (*β* = 0.18, *p* = 0.001, *R*^2^ = 0.03), reducing public activities (*β* = 0.35, *p* *<* 0.001, *R*^2^ = 0.13), and helping others to prevent the epidemic (*β* = 0.27, *p* *<* 0.001, *R*^2^ = 0.07). However, age had less influences on health habits for the epidemic prevention, and had greater influences on reducing public activities and helping others to prevent the epidemic.

### 3.3. Relationships among COVID-19 Knowledge, Affective Attitude, and Preventive Behavior

Table 6 shows that gender and age could explain 9% of the variation in the epidemic prevention hygiene habits. After controlling for the variables of gender and age, COVID-19 knowledge and positive/negative affective attitudes could increase 7% of the variation in the epidemic prevention hygiene habits, of which only psychological wellbeing had a significant explanatory power for the epidemic prevention hygiene habits (*β* = 0.25, *p* *<* 0.001), showing that a higher psychological wellbeing was correlated with better epidemic prevention hygiene habits.

Gender and age also had significant explanatory power for reducing public activities and helping others to prevent the epidemic, respectively explaining 14% and 8% of the behavioral variation. After controlling for the variables of gender and age, COVID-19 knowledge and positive/negative affective attitudes could increase 5% of the variance in reducing public activities and 9% of the variance in helping others to prevent the epidemic, with psychological wellbeing and emotional wellbeing having significant explanatory power for reducing public activities (*β*_(psychological wellbeing)_ = 0.18, *p* = 0.009; *β*_(emotional wellbeing)_ = −0.13, *p* = 0.02) and helping others to prevent the epidemic (*β*_(psychological wellbeing)_ = 0.20, *p* = 0.004; *β*_(emotional wellbeing)_ = −0.13, *p* = 0.03).

## 4. Discussion

This study mainly discussed COVID-19 knowledge, affective attitudes, and preventive behaviors of the Taiwanese public. From the perspective of COVID-19 knowledge, although the questions were easy for the participants, the purposes of knowledge measurement were not to distinguish the participants’ knowledge, but to understand whether they had basic knowledge about the COVID-19 disease in terms of pathology, symptoms, and prevention. According to the results, the participants, regardless of gender or age, had a good understanding of COVID-19, in contrast to previous studies revealing relatively less knowledge about COVID-19 disease transmission [38,39,40], but similar to Kasemy et al.’s research [41]. Moreover, there was no significant difference in knowledge as a function of education, age, or gender, unlike prior studies [13,41,42]. It was inferred that most participants had higher education levels, as there were only 9.71% participants without a bachelor’s degree; in addition, the general public’s awareness of the information provided by the TCDC was facilitated by its availability on various social media platforms and TV channels. In terms of positive affective attitudes, participants’ emotional wellbeing was generally lower amid the pandemic. Emotional wellbeing includes positive emotions such as personal pleasure, happiness, and satisfaction with life, as well as pain avoidance [43,44,45]. However, individuals encountered many inconveniences in their daily life, such as travel, socializing with relatives and friends, daily work, and recreational activities, which were all restricted to varying degrees during the pandemic [46], thus leading to the sacrifice of some pleasant experiences [47], further lowering emotional wellbeing. This also reflected that participants had relatively negative feelings and attitudes toward their personal external life during the pandemic.

The decline in emotional wellbeing and increase in negative perceptions of life should receive more attention in younger groups. Wang et al. [48] claimed that, during the COVID-19 pandemic, adolescents not only had to face the typical stress of adolescence growth, but also the stress of COVID-19 concerns, providing obstacles and dilemmas in meeting needs, acquiring abilities, and building relationships with others, and further increasing the likelihood of maladjustment and a reduction in personal wellbeing. Similarly, Cheng et al. [49] also pointed out adolescents are not fully matured psychologically or physically; thus, they are more likely to experience negative emotions when faced with a major stressful event such as the COVID-19 outbreak.

According to this study, the effect of age on emotional attitudes was very small, which may be because the impact of the COVID-19 outbreak during the study period in Taiwan was not severe compared with other countries. The severe COVID-19 outbreak (i.e., level 3 alert) in Taiwan actually started in May 2021, when the government began to fully implement stricter epidemic prevention restrictions (e.g., requiring comprehensive masks when going out, the closure of leisure and entertainment places, the cessation of indoor use in the catering industry, and restrictions on weddings, funerals, festivals, and religious activities). At the time of this study, the COVID-19 pandemic may have had little impact on the Taiwanese public’s happiness, thus reducing the effect of participants’ age. After Taiwan raised the epidemic alert to the third level, the epidemic prevention measures may have had a more severe impact. Therefore, future studies can explore adolescents’ stressors, stress responses, and the changes in emotional states across the entire COVID-19 pandemic.

In this study, the participants’ sociodemographic status showed significant differences with respect to COVID-19 preventive behaviors. Females were better than males at cooperating with epidemic prevention hygiene habits, reducing public activities, and helping others to prevent the epidemic, indicating a generally higher risk awareness of the disease; thus, they took COVID-19 more seriously and were more proactive with respect to preventive behaviors [6,7,50]. Moreover, the younger participants were less inclined to take COVID-19 preventive behavioral measures, which may be because younger groups are more prone to “optimistic bias”, making them feel less vulnerable than others [51]. This misjudgment of risk would cause a lower perception of disease risk than older adults, thus lowering adherence to the preventive behaviors [52]. It is suggested that future studies explore the optimism bias and perceived susceptibility to disease; in practice, it is recommended to increase the risk awareness of susceptibility to COVID-19 among the younger groups to prevent infection.

On the relationships among COVID-19 knowledge, emotional attitudes, and preventive behaviors, this study discovered that the key to determining whether the participants engaged in preventive health behaviors was affective attitudes, especially positive affective attitudes, rather than COVID-19 knowledge, consistent with the previous findings that COVID-19 knowledge does not necessarily induce individuals’ preventive behaviors [19], and that attitudes about COVID-19 may be the key to driving individuals’ actions [21]. However, it is worth noting that the influence of knowledge in this study was not obvious, which may because most of the participants in this study had a high COVID-19 knowledge level. Nonetheless, this study highlighted the importance of emotional attitudes; among COVID-19 knowledgeable groups, people with higher personal emotional wellbeing were less inclined to take preventive behaviors such as reducing public activities and helping others to prevent the epidemic. This may be because preventive health behavior measures that reduce public activities, such as going out for gatherings and meals, may cause a decrease in personal pleasant experiences [47], whereas helping others to prevent the epidemic may make individuals acknowledge their losses, thereby increasing negative emotions [44]. In other words, when people build their wellbeing primarily on personal emotional perceptions of experiencing pleasure and avoiding pain, it may be detrimental to the adoption of these preventive health behaviors.

In contrast to emotional wellbeing, individuals with higher psychological wellbeing tended to be more inclined to take COVID-19 preventive behaviors. Psychological wellbeing involves the belief in self-development, whereby happiness is based on personal growth and becoming a better person [43,44,45]. From the perspective of public health policy, preventive behaviors for infectious diseases not only help reduce the chance of infection in oneself, but also create benefits of reducing the risk of infection in others; thus, the adoption of preventive behaviors can satisfy both personal and public interests [53,54]. In other words, the adoption of preventive behaviors can help individuals make contributions to defending community infection, as well as to their needs to become a better person. Therefore, when people build their wellbeing primarily on the psychological aspect of being a better person, they may be more inclined to take preventive behaviors against COVID-19.

This study had several limitations. Firstly, COVID-19 prevention behaviors in this study were a result of participants’ subjective self-reports, and their reported behaviors may not be consistent with their actual behaviors. Secondly, the duration of the investigation was before the third level of Taiwan’s pandemic alert; hence, its findings may be different from the situation after the alert was upgraded. Thirdly, due to its cross-sectional design to investigate the relationships among COVID-19 knowledge, attitudes, and behaviors, and the data being analyzed a single time point, the interpretation and inference of causal relationships in this study must be interpreted with caution. Fourthly, the participants’ age in this study ranged from 15 to over 65 years old, but most of them were under 30, which may be because the use of online questionnaires through social networks limited the diversity of participants. Therefore, future studies may need to gather more diverse background groups through multiple channels. Despite this limitation, the WHO [8] claimed that the KAP model can help to understand the relationships among people’s knowledge, attitudes, and behaviors on a specific issue, which can serve as an important reference for the evaluation of related programs or intervention measures. Thus, we believe that KAP studies related to the COVID-19 pandemic can contribute significantly. Lastly, this study did not extensively explore other cognitive factors associated with COVID-19 behaviors, such as perceived barriers or the new media health literacy level, which could influence public knowledge, including seeking information, processing information, or evaluating information. Moreover, this study did not extensively examine affective factors associated with COVID-19 behaviors, such as strengthening factors associated with resilience. Nevertheless, the findings of this paper provide valuable empirical evidence for organizations to support strategies and pre-emptive plans.

## 5. Conclusions

Although COVID-19 knowledge does not necessarily induce people to take preventive behaviors, this does not mean that public disease knowledge is not important. Knowledge is the foundation of behavior and the reason for taking action. When the government and relevant authorities educate people about COVID-19-related diseases and infections, they should also think about how to induce changes in the public from “empty talk” to “do it now”. In addition, it is important to trigger the susceptibility of younger groups to COVID-19, especially adolescents. Using moral persuasion rather than threatening language may help to promote preventive behaviors; for example, phrases such as such as “we need you” and “you can help society” can be used to improve public compliance with COVID-19 preventive behaviors and measures. Nevertheless, when requesting the public to follow preventive measures, the government should also pay attention to the people’s emotional wellbeing with respect to their negative perception of life amid the COVID-19 pandemic. Hence, to prevent other mental health disorders derived from the epidemic, especially in teenagers who are immature physically and mentally, mental health requires more attention. We suggest that relevant authorities or education departments should develop relevant propaganda or educational interventions from the perspective of morality or social cohesion to enhance people’s willingness to take preventive actions in the future. How to provide mental health assistance to younger groups amid the pandemic, especially with respect to social distance protocols, also needs to be explored in future research. It is suggested that future studies can build on the present study to further identify the key positive factors affecting personal strategies to improve self-resilience during the COVID-19 pandemic; in addition, more effective psychological interventions should be explored to help and strengthen the psychological resilience of vulnerable groups such as medical patients and those financially impacted by the epidemic.

## Figures and Tables

**Table 1:** Participant composition.

Participants	*n*	Percentage	Cumulated Percentage
**Gender**			
Male	136	40.00%	40.00%
Female	204	60.00%	100.00%
**Education**			
Associate degree	33	9.71%	9.71%
Bachelor’s	227	66.76%	76.47%
Master’s or doctoral	80	23.53%	100.00%
**Occupation**			
Student	212	62.35%	62.35%
Nonstudent	128	37.65%	100.00%
**Age**			
15–20	120	35.29%	35.29%
21–30	136	40.00%	75.29%
31–40	48	14.12%	89.41%
41–50	16	4.71%	94.12%
51–66	20	5.88%	100.00%

**Table 2:** COVID-19 negative affective attitude scale.

Items	Commonality	Factor Analysis		Explained Variations	Reliability
		Negative Self-Perception	Negative Perception of Life		
1. I find it hard to do anything.	0.74	0.85	0.13	49.97	0.90
2. I feel depressed.	0.71	0.81	0.24		
3. I cannot concentrate when I am doing things.	0.65	0.80	0.07		
4. Even with the help of relatives and friends, I still cannot get away my worries.	0.67	0.80	0.17		
5. I do not sleep well.	0.53	0.71	0.15		
6. I think my life is a failure.	0.53	0.64	0.34		
7. I feel sad.	0.56	0.63	0.40		
8. I feel scared.	0.53	0.63	0.36		
9. I enjoy the enjoyments of my life (reverse).	0.75	0.16	0.85	13.28	0.78
10. I am happy (reverse).	0.74	0.20	0.84		
11. I am hopeful for the future (reverse).	0.57	0.20	0.72		

**Table 3:** COVID-19 preventive behavior scale.

Items	Commonality	Factor Analysis			Explained Variations (Reliability)
		Epidemic Prevention Hygiene Habits	Reducing Public Activity	Helping Others	
1. I specifically pay attention to respiratory hygiene and cough etiquette.	0.75	0.81	0.13	0.26	44.04 (0.86)
2. When entering and exiting enclosed spaces, I wear a mask autonomously.	0.67	0.79	0.20	0.08	
3. I wash my hands more often than ever.	0.66	0.78	0.21	0.03	
4. I try to avoid coughing when there are people.	0.62	0.75	0.18	0.18	
5. I specifically pay attention to the cleanliness and ventilation of the environment at home.	0.57	0.64	0.30	0.26	
6. I reduce the number of times that I go out for shopping.	0.79	0.16	0.86	0.18	12.74 (0.83)
7. I take less public transportation.	0.62	0.15	0.75	0.18	
8. I avoid entering and exiting enclosed spaces such as libraries, fitness centers, theaters, and movie theaters.	0.68	0.33	0.74	0.15	
9. I cancel or postpone dinners with friends.	0.58	0.21	0.73	0.10	
10. If I do not need to use surgical masks, I am willing to give the masks to those who need.	0.75	0.13	0.12	0.85	11.57 (0.83)
11. If the social welfare organization has difficulties in epidemic prevention, I am willing to donate or provide other forms of assistance.	0.73	0.13	0.20	0.82	
12. If others have needs in epidemic prevention, I am willing to help others to take epidemic prevention measures.	0.78	0.27	0.20	0.82	

**Table 4:** COVID-19 knowledge test.

Question	Answer	Correctness Rate
1. According to current reports, the first confirmed case of COVID-19 was diagnosed in Wuhan, China.	T	94.12%
2. COVID-19 spreads through human-to-human transmission routes.	T	100.00%
3. The incubation period for infection with COVID-19 is up to 14 days, and the average is 5 days.	T	79.12%
4. Taiwan lists COVID-19 as the fifth national notifiable infectious disease.	T	75.59%
5. Regarding COVID-19, countries have different testing methods. Currently, Taiwan adopts RT-PCR testing, which is collected and submitted through throat swabs.	T	84.71%
6. Coronaviruses are easily isolated by tissue culture.	F	62.94%
7. The novel coronavirus belongs to Coronavirinae (CoV) and is an important pathogen causing human and animal diseases.	T	94.71%
8. The animal hosts of the coronavirus include bats (the largest), pigs, cattle, cats, dogs, and ferrets, and there are sporadic reports of cross-species transmission.	T	87.94%
9. CoV is a group of viruses with a mantle, round in appearance, with crown-like protrusions can be seen under an electron microscope (hence, the name).	T	91.47%
10. Whether the novel coronavirus that causes COVID-19 has an animal host remains to be researched and confirmed.	T	84.12%
11. The disease can be treated with conventional antiviral drugs.	F	68.82%
12. Most confirmed cases of COVID-19 are severe.	F	76.47%
13. After being tested for COVID-19, you need to stay home until the results are notified.	T	93.24%
14. The Ministry of Education (MoE) stipulates that a school should be closed for 2 weeks if there are two COVID-19 confirmed cases in the school.	T	91.18%
15. If symptoms develop within 14 days of direct contact with a suspected case, you should consult the nearest public health unit.	T	95.29%
T: True, F: False		

**Table 5:** Gender differences in COVID-19 knowledge, affective attitude, and preventive behavior.

Variables	Male		Female		*t*	*p*
	*Mean*	*SD*	*Mean*	*SD*		
COVID-19 knowledge	12.94	1.30	12.80	1.37	0.96	0.34
Positive affective attitudes						
Emotional wellbeing	2.41	0.91	2.31	0.96	0.98	0.33
Psychological wellbeing	3.59	0.77	3.64	0.67	0.65	0.51
Social wellbeing	3.06	0.80	3.06	0.80	0.06	0.95
Negative affective attitudes						
Negative self-perception	2.23	0.80	2.14	0.72	1.16	0.25
Negative perception of life	2.79	0.76	2.92	0.77	−1.05	0.13
Preventive behavior						
Prevention hygiene habits	4.10	0.65	4.43	0.54	−5.08 **	*p* < 0.01
Reducing public activities	3.48	0.89	3.76	0.84	−3.00 **	*p* < 0.01
Helping others to prevent the epidemic	3.82	0.76	3.99	0.79	−1.97 *	*p* < 0.05

* *p <* 0.05; ** *p <* 0.01

**Table 6:** Hierarchical regression analysis of COVID-19 knowledge and positive/negative affective attitudes on preventive behaviors.

	Epidemic Prevention Hygiene Habits			Reducing Public Activities			Helping Others to Prevent the Epidemic		
	*β*	*t*	*p*	*β*	*t*	*p*	*β*	*t*	*p*
Pattern 1									
Gender									
Female	0.25	4.74 ***	*<*0.001	0.12	2.32 *	0.02	0.07	1.40	0.16
Age	0.15	2.78 **	0.006	0.34	6.63 ***	*<*0.001	0.26	4.83 ***	*<*0.001
	*F*_(2,337)_ = 17.02 ***			*F*_(2,337)_ = 27.06 ***			*F*_(2,337)_ = 13.75 ***		
	*R =* 0.30, *R*^2^ = 0.09			*R =* 0.37, *R*^2^ = 0.14			*R =* 0.28, *R*^2^ = 0.08		
Pattern 2									
Gender									
Female	0.23	4.56 ***	<0.001	0.11	2.27 *	0.02	0.08	1.47	0.14
Age	0.09	1.74*	0.08	0.28	5.39 ***	<0.001	0.19	3.50 ***	<0.001
Knowledge	−0.08	−1.49	0.14	−0.01	−0.13	0.90	−0.02	−0.36	0.72
Emotional wellbeing	−0.09	−1.47	0.14	−0.13	−2.29 *	0.02	−0.13	−2.19 *	0.03
Psychological wellbeing	0.25	3.76 ***	<0.001	0.18	2.65 **	0.008	0.20	2.93 **	0.004
Social wellbeing	−0.05	−0.83	0.41	−0.11	−1.76 *	0.08	0.05	0.77	0.44
Negative self-perception	−0.04	−0.72	0.47	−0.01	−0.13	0.90	−0.03	−0.51	0.61
Negative perception of life	−0.01	−0.08	0.94	−0.05	−0.65	0.52	−0.11	−1.53	0.13
	*F*_(6,331)_ = 8.13 ***			*F*_(6,331)_ = 9.73 ***			*F*_(6,331)_ = 8.29 ***		
	*R =* 0.41, *R*^2^ = 16			*R =* 0.44, *R*^2^ = 19			*R =* 0.41, *R*^2^ = 17		
	Δ*R*^2^ = 0.07, *p <* 0.001			Δ*R*^2^ = 0.05, *p =* 0.002			Δ*R*^2^ = 0.09, *p <* 0.001		
Δ*R^2^*: Change in *R*^2^									

* *p <* 0.05; ** *p <* 0.01; *** *p <* 0.01. *Beta*: standardized coefficient.

## Data Availability

The data presented in this study are available on request from the corresponding author.
